# Unveiling Hidden Allies: In Silico Discovery of Prophages in *Tenacibaculum* Species

**DOI:** 10.3390/antibiotics13121184

**Published:** 2024-12-05

**Authors:** Carolina Ramírez, Jaime Romero

**Affiliations:** Laboratorio de Biotecnología de Alimentos, Instituto de Nutrición y Tecnología de los Alimentos (INTA), Universidad de Chile, El Líbano 5524, Santiago 7830489, Chile; carolina.ramirez.saavedra@gmail.com

**Keywords:** Tenacibaculosis, phage therapy, *Tenacibaculum*, endolysin, aquaculture

## Abstract

Tenacibaculosis, caused by *Tenacibaculum* species, is a significant disease in aquaculture, leading to high mortality and economic losses. Antibiotic treatment raises concerns about resistance, making phage therapy an interesting alternative. Analyzing phage traces in *Tenacibaculum* genomes is crucial for developing these bacteriophage-based strategies. Methods: We assessed the presence of prophages in 212 *Tenacibaculum* genomes/assemblies available in the NCBI repository, comprising several species and global locations, using the PHASTEST program. Then, we focused on those regions classified as intact, evaluating the most common phages found using VICTOR. The protein of interest discovered in the prophages was evaluated using the ProtParam, DeepTMHMM, InterPro, and Phyre2 tools. In addition, we evaluated the presence of antiphage defense systems in those genomes with intact prophages using the DefenseFinder tool. Results: We identified 25 phage elements in 24 out of the 212 *Tenacibaculum* genomes/assemblies analyzed, with 11% of the assemblies containing phage elements. These were concentrated in *T. maritimum* and *T. mesophilum*, which harbored 10 and 7 prophage regions, respectively. Of the identified elements, six were classified as intact, including four in *T. maritimum*, with the most common phages belonging to the *Pippivirus* and *Siphoviridae* families. Bioinformatic analysis showed that the putative endolysin is a stable protein of 432 amino acids and 49.8 kDa, with three transmembrane helices and a CHAP domain, structurally similar to the CHAP lytic domain of *S. aureus* bacteriophage K. Conclusions: Key prophage elements in *Tenacibaculum*, especially in *T. maritimum*, show promise for phage therapy against tenacibaculosis, supporting sustainable, antibiotic-free treatments in aquaculture.

## 1. Introduction

*Tenacibaculum* is a genus of Gram-negative bacteria belonging to the *Flavobacteriaceae* family in the phylum *Bacteroidetes*, commonly found in marine environments and affecting various aquatic organisms, primarily fish. This bacterium is known for causing skin diseases in fish, such as tenacibaculosis, characterized by skin lesions, ulcers, and tissue necrosis. This pathogen is a significant concern in aquaculture, particularly in the farming of marine fish, as it impacts both fish health and the profitability of production [1].

The genus *Tenacibaculum* comprises more than 30 distinct species, of which eight have been linked to diseases in fish [2]. Tenacibaculosis, the disease caused by this genus, affects a variety of fish species, including Atlantic salmon (*Salmo salar*), European seabass (*Dicentrarchus labrax*), sole (*Solea senegalensis*), and turbot (*Scophthalmus maximus*) [3,4,5]. The majority of existing studies on *Tenacibaculum* species link disease in marine fish to infections caused by *Tenacibaculum maritimum* [2,3].

*Tenacibaculum maritimum* was formally described by Suzuki in 2001 [6], following its initial isolation from fish suffering from skin diseases. Prior to this definitive classification, bacteria within this genus were commonly categorized as part of the “Flexibacter group” due to their connection with the genus *Flexibacter*, which also belongs to the *Flavobacteriaceae* family. Since then, mortality events linked to *Tenacibaculum* have been on the rise and spreading worldwide [7]. In 2023, mortality rates among salmon in the grow-out phase in Norway reached unprecedented levels, largely due to disease syndromes like winter ulcer disease and complex gill disease. Winter ulcer disease, primarily caused by Moritella viscosa and *Tenacibaculum* species, represents the most significant bacterial health and welfare challenge in Norwegian aquaculture. Furthermore, Spilsberg 2022 [8] reported a strong association between the predominant presence of *T. finnmarkense* and the development of severe cranial lesions in Atlantic salmon smolts that had recently been transferred to the sea. Meanwhile, in Chile, the second-largest producer of salmonids after Norway, the health authority has recently reported that, in terms of infectious mortality in Atlantic salmon, tenacibaculosis ranks second, accounting for 37.9% of cases. Compared to 2022, tenacibaculosis has seen an increase of 9% [9].

Currently, no widely available and effective commercial vaccines exist for controlling *Tenacibaculum maritimum*, the primary causative agent of tenacibaculosis in marine fish. The only commercial vaccine available is ICTHIOVAC^®^ TM, an inactivated injectable vaccine for tenacibaculosis in turbot [10]. Due to the bacterium’s complexity and the variable conditions in aquaculture, infection prevention and management typically depend on good husbandry practices, environmental controls, and, in some cases, antibiotic treatments.

Bacteriophages have been proposed as antimicrobial agents to combat pathogenic bacteria in various fields, including aquaculture. Research has highlighted numerous examples of phage applications aimed at controlling pathogenic vibrios, which pose significant challenges in aquaculture [11,12,13]. Additionally, the bacteriophage-based product Curtus has been developed specifically to address *Yersinia* infections in aquaculture settings, offering a targeted and eco-friendly approach to managing this pathogen and improving fish health [14]. Recently, Tsertou [15] reported the isolation and characterization of a bacteriophage against *Tenacibaculum larymnensis* sp. nov., a novel species within the *Tenacibaculum* genus, which was isolated from a commercial fish hatchery in Greece.

In this context, this research aims to deepen our understanding of the interactions between *Tenacibaculum* host bacteria and their corresponding bacteriophages through genomic analysis, with a particular focus on the bacterial defense mechanisms [16]. By elucidating these relationships, the study endeavors to identify novel insights that may contribute to the formulation of effective strategies for the control of these pathogenic bacteria.

## 2. Results

We identified 25 phage elements in 24 out of the 212 Tenacibaculum genomes/assemblies analyzed, representing approximately 11% of the total assemblies. These elements were predominantly found in two species, *T. maritimum* and *T. mesophilum*, which harbored 10 and 7 prophage regions, respectively (Table 1). Notably, this distribution is not due to a larger number of strains analyzed for these species; 40 assemblies of *T. maritimum* and 10 of *T. mesophilum* were examined. In contrast, *T. finnmarkense*, the most represented species in the dataset with 129 assemblies, revealed only a single assembly containing phage-related elements.

Based on the classification provided by PHASTEST, of the 25 identified phage elements, 6 were categorized as intact, 8 as questionable, and 11 as incomplete. Notably, four of the intact phages are found in *T. maritimum*. Among the intact prophage regions, the most frequently identified phages include two associated with Flavobacterium—vB_FspM_pippi8-1 (NC_048830) and vB_FspM_lotta8-1 (NC_048829)—and two associated with Cellulophaga—phi19:1 (NC_021799) and phi39:1 (NC_021804). The former two belong to the family *Pippivirus*, whereas the latter two are classified under *Siphoviridae*.

**Figure 1:**
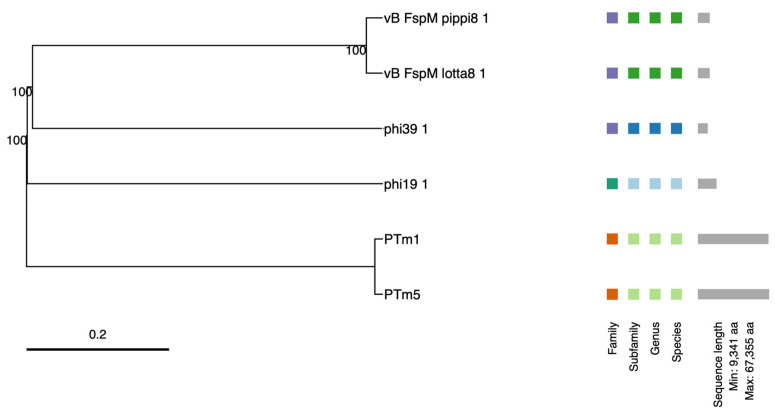
Phylogenetic analysis of the 4 prophages found as the most common in the regions classified as intact in this study and the phages described for *Tenacibaculum* in previous studies at the amino acid level using VICTOR. The Genome BLAST Distance Phylogeny tree (GBDP) was derived using the D6 formula and yielded an average support of 100%. The OPTSIL clustering produced 3 clusters at family level and 4 clusters at species, genus, and subfamily levels, these clusters are represented by different colors.

For the phylogenetic analysis of the most common phages from intact regions and the previously described *Tenacibaculum* phages (PTm1 and PTm5), the OPTSIL clustering results (Figure 1) show that these phages cluster differently. The prophages found in this study form distinct groups from those described in earlier research. Specifically, three clusters are observed at the family level and four clusters at the subfamily, genus, and species levels. Additionally, the previously described *Tenacibaculum* phages have notably longer sequences compared to those identified in this study.

**Figure 2:**
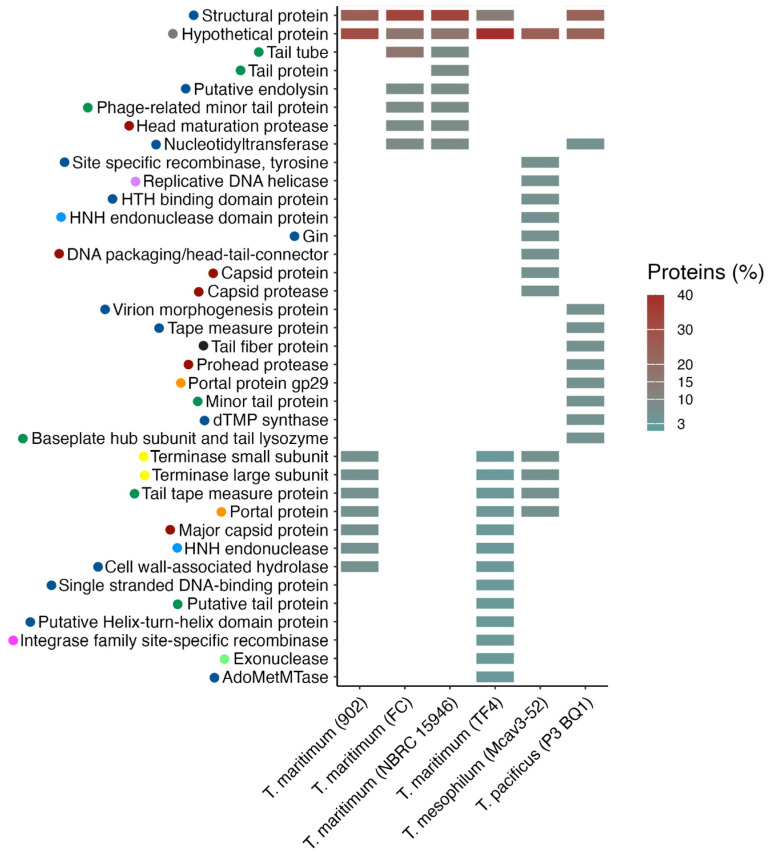
Coding sequences identified in prophages classified as intact according to PHASTEST in *Tenacibaculum* strains. CDS were grouped in line with the BLAST description and presented as a percentage of proteins per strain. The colors contained in the circles on the left side of the protein description indicate the type of protein: phage-like proteins are highlighted in blue, tail proteins in green, head proteins in red, terminase in yellow, endonuclease in light blue; portal proteins in orange; the hypothetical protein in gray; fiber protein in dark gray; exonuclease in light green; integrase in pink, and DNA helicase in light pink.

A total of 101 phage-associated CDS were identified among the 6 intact prophages (Supplementary Table S1). These were grouped according to their description in the BLAST hit (Figure 2), which highlighted that most of the proteins present in the different *Tenacibaculum* strains correspond to structural or hypothetical proteins (ranging from 14 to 39%). The coding sequences identified belong to phages of the class *Caudoviricetes*, except for one, which is unclassified (Supplementary Table S1). Therefore, it is possible to note several tail-associated proteins (tail tube, tail protein, and phage-related minor tail protein, among others). Other relevant components were also found, such as terminase and endolysin, which are involved in the phage DNA packaging and infection-related process, respectively (Figure 2).

A putative endolysin emerged as a protein of interest in this analysis. It was present in two of the six strains with intact classified prophages, *T. maritimum* strain FC and NBRC 15946. According to the BLAST hit, this protein showed homology with an endolysin described in *Streptomyces* phage TG1 (NC_018853). ProParam analyses showed that the putative endolysin, a protein of 432 amino acids in length, has a molecular weight of 49.8 kDa and an instability index of 34.38, which classifies the protein as stable. In addition, transmembrane helix prediction showed three TMhelix sites in the putative endolysin, positioned between amino acids 12–29, 48–66, and 100–109. Moreover, the results of the InterPro analysis showed the presence of the CHAP (cysteine, histidine-dependent amidohydrolases/peptidases) domain at amino acid positions 158–246 of the putative endolysin (Supplementary Figure S1). Structural prediction and homology modeling of the putative endolysin using the Phyre2 server showed that the CHAP domain had similar folding topology to that of the CHAP lytic domain of endolysin k from *Staphylococcus aureus* bacteriophage k, with 98.37% confidence but with a low percentage identity of 19% (Supplementary Figure S2).

**Figure 3:**
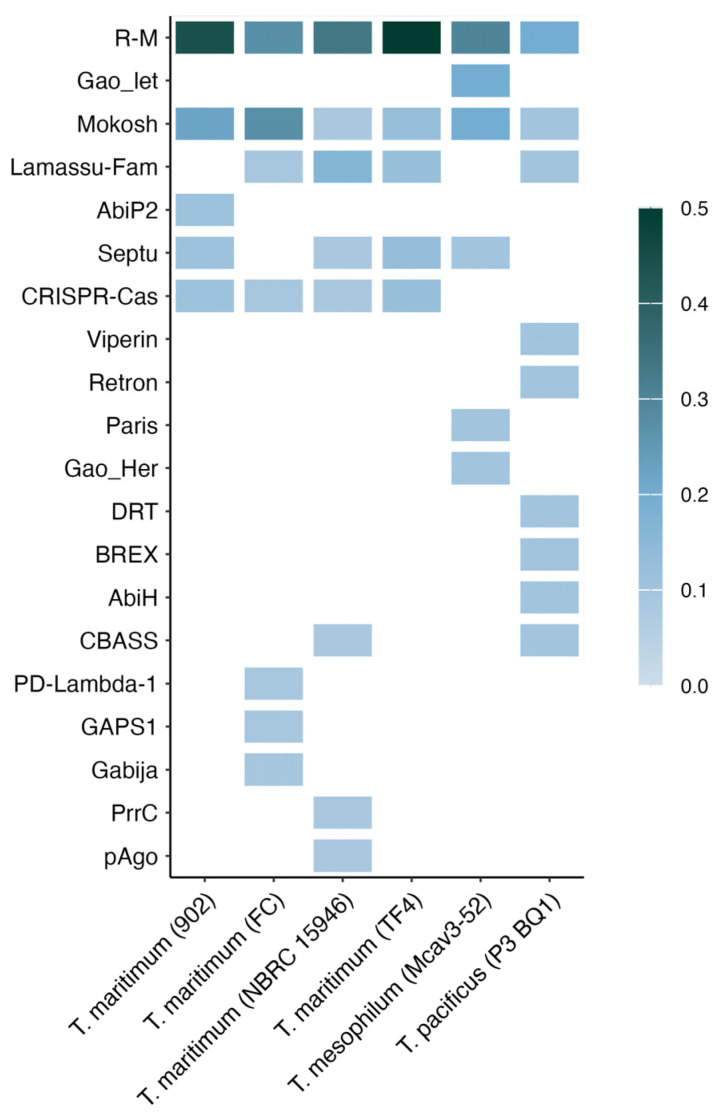
Defense systems discovered in *Tenacibaculum* strains that presented prophage regions classified as intact. The colors represent the proportion of defense system types included in the genome.

In addition, we evaluated the antiphage systems present in *Tenacibaculum* genomes that had regions with prophages classified as intact. The results showed an average of 10 defense systems per strain. The systems present in the six genomes were the restriction-modification (R-M) and Mokosh systems, whereas CRISP-Cas was only present in the four genomes of *T. maritimum* (Figure 3). The results also highlighted that, in general, the R-M system was present in greater proportions in the genomes of the strains (Supplementary Table S2).

## 3. Discussion

The distribution of prophage elements observed in *Tenacibaculum* species reveals a selective presence of phage elements, with notable concentrations in *T. maritimum* and *T. mesophilum*. This distribution pattern, where prophage elements are concentrated in specific species rather than in the species with the highest representation in the dataset (e.g., *T. finnmarkense*, which showed minimal prophage presence despite its 129 assemblies), suggests a unique evolutionary or ecological adaptation in *T. maritimum* and *T. mesophilum*. This trend is consistent with studies in other bacterial genera, where prophage presence varies significantly between species within the same genus, often influenced by niche-specific factors or host interactions [17]. Similar results were described by [18,19]. In *Mycobacterium* strains [18], the distribution of prophages demonstrates notable variability across taxa, with most strains within five major groups lacking prophages entirely, in contrast to the *M. abscessus-chelonae* complex, where approximately 75% of strains contain at least one prophage. This pattern reflects the significant role of ecological and evolutionary pressures in shaping prophage retention and loss within specific bacterial lineages, suggesting that niche specialization or host interactions may influence prophage prevalence in certain taxa more than others [20]. The low prophage prevalence in *T. finnmarkense* might suggest reduced exposure to phage interactions or differing survival strategies in its ecological niche. Overall, this distribution underlines how prophage content can reflect bacterial adaptation and highlights *T. maritimum* and *T. mesophilum* as potential candidates for exploring phage-based control strategies in aquaculture due to their higher prophage load and potential phage susceptibility.

The diversity observed among *Tenacibaculum* phages, with distinct clusters at the family, subfamily, genus, and species levels, reflects a broad genetic variability that has also been reported in other bacterial genera with complex prophage populations. For instance, *Staphylococcus aureus* exhibits a wide range of prophages that form unique phylogenetic clusters, with some strains harboring multiple distinct prophages that contribute to genetic and functional diversity across strains, including virulence factor variation [21]. In *Mycobacterium*, strains in the *M. abscessus-chelonae* complex show high prophage diversity, with unique clusters across subgroups that support varied adaptations within environmental or host-associated niches [18]. This genetic diversity in *Mycobacterium* and other bacteria, as observed in *Tenacibaculum*, indicates that prophage diversity is often driven by ecological adaptation, host-specific interactions, or environmental pressures, which can lead to the formation of distinct phylogenetic groups [17,20,22].

Bacteriophages are abundant in the environment, particularly in seawater, and have been explored as an alternative to chemical antimicrobials in aquaculture [12,13]. Despite their potential, the use of phages in aquaculture poses risks, including the emergence of new virulent bacteria due to lysogenic conversion, where temperate phages can introduce virulence genes into bacterial genomes, as observed in *Vibrio cholerae* and *V. harveyi* [23,24]. Additionally, phages can facilitate horizontal gene transfer through transduction, potentially spreading virulence factors to non-pathogenic strains or even to different bacterial species [23,25]. Although lytic phages may exert selective pressure that leads to phage-resistant bacteria, which are often thought to have reduced virulence, evidence suggests that resistance does not always reduce pathogenicity, raising further concerns about the use of phages as antimicrobials [26,27]. However, phage-derived endolysins do not carry these risks, as they lack the capacity for lysogenic conversion or horizontal gene transfer, making them a safer alternative in antimicrobial strategies. Moreover, unlike endolysins, bacteriophages must overcome bacterial defense mechanisms specifically designed to block the phage replication cycle, such as antiphage defense systems (e.g., CRISPR-Cas, restriction-modification systems, and abortive infection systems).

A recent pangenome analysis revealed new bacterial defense systems that challenge phage effectiveness [28]. Understanding these defenses and their limitations is crucial for advancing phage applications [29]. In this study, the most commonly detected antiphage defense mechanisms were the restriction-modification (R-M) and Mokosh systems, which were present across multiple genomes. In contrast, the CRISPR-Cas system was found exclusively in the four genomes of *T. maritimum* (Figure 3). The restriction-modification (R-M) system uses two enzymes: a restriction endonuclease and a methyltransferase. The methyltransferase protects bacterial DNA by adding methyl groups to specific sites, whereas unmethylated foreign DNA, like phage DNA, is recognized by the restriction enzyme and cut, effectively degrading it [30]. Mokosh systems were recently discovered; unlike other bacterial immune systems, such as CRISPR-Cas, Mokosh does not rely on a memory of past infections but instead offers an immediate defensive response through the degradation of the phage’s genetic material or interference with essential phage processes [31]. Mokosh includes an RNA helicase domain (COG1112 family) and a PLD family nuclease. In type I Mokosh, the RNA helicase and PLD nuclease are encoded by separate genes, with an additional serine–threonine kinase (STK) domain. In type II, these domains are combined within a single gene, with the RNA helicase at the N-terminus and the PLD nuclease at the C-terminus. In *T. larymnensis*, Mokosh type II, as described by [15], includes the mkoC gene, featuring an RNA helicase at the N-terminus and a PLD nuclease as the effector domain.

CRISPR-Cas is another antiphage defense mechanism. It functions as an adaptive immune system by capturing segments of phage genomes, known as spacers, and storing them in a repeat–spacer array within a CRISPR locus. During a later infection, this array is transcribed into CRISPR-RNA (crRNA) [32,33]. These repeat–spacer sequences then guide endonucleases, like Cas9, to locate and cleave matching sequences in the invading phage genome. CRISPR-Cas systems are notably enriched in certain bacterial pathogens, likely as a response to selective pressures in complex environments like the mucosa [33,34]. This immune mechanism may help pathogens evade phages while retaining virulence, particularly when interacting with the host’s mucosal layers. The presence of mucosa appears to drive a diversification of bacterial immune strategies, as pathogens must balance an effective phage defense with the need to avoid modifications that could hinder mucosal colonization. Studies indicate that exposure to mucosal components like mucin can enhance bacterial susceptibility to phages, as seen in *Flavobacterium columnare*, *Aeromonas* species, *Clostridium difficile*, and *Escherichia coli* [34,35].

Recently, Wu 2024 noted that certain defense systems that are negatively associated in *E. coli* show synergistic effects and frequently co-occur in other species, indicating that bacterial immune repertoires are shaped more by selective pressures from host-specific phages than by negative interactions between systems. These findings suggest that bacterial defense systems are compatible and often work together, enabling bacteria to develop adaptable strategies against phage attacks.

Endolysins, or lysins, are enzymes produced by bacteriophages during the later stages of their infection cycle. These proteins target and break down the peptidoglycan (PG) in bacterial cell walls, compromising the structural integrity of the cell. This degradation leads to cell death, typically through osmotic lysis, as the weakened cell wall can no longer withstand the internal pressure, causing the bacterial cell to burst [36,37]. Studies on bacteriophages have enhanced our understanding of endolysins’ role in the phage lytic cycle and their potential as antimicrobial agents. In aquaculture, endolysins show promise as an effective alternative for combating bacterial infections [38]. Their mechanism for breaking down the bacterial cell wall reduces the chances of resistance emerging and is regarded as safe for eukaryotic organisms [37].

Our analysis identified an endolysin similar to LysK from *Staphylococcus aureus* bacteriophage K, including its CHAP domain (cysteine, histidine-dependent amidohydrolase/peptidase domain) [39]. N-acetylmuramoyl-L-alanine amidase is an enzyme that plays a crucial role in breaking down bacterial cell walls. It specifically targets the bond between N-acetylmuramic acid (a component of the peptidoglycan layer in bacterial cell walls) and L-alanine in the peptide chain. By cleaving this bond, the enzyme contributes to the degradation of the peptidoglycan structure, leading to cell wall disruption and, ultimately, bacterial cell lysis [40].

Phage lysins could be very useful in the control of aquaculture pathogens, like vibrios. The enzyme LysVPp1 effectively lysed 9 out of 12 *Vibrio parahaemolyticus* strains, displaying a broader range of action than its associated phage VPp1, which was active against only three strains [41]. Similarly, LysVpKK5 exhibited potent lytic activity against the VPATCC-17802 strain, even though the original VpKK5 phage could not infect it [42,43]. Furthermore, several vibriophage endolysins have shown lytic activity on sensitized cells of other Vibrio species, including *V. mimicus*, *V. anguillarum*, *V. harveyi*, and *V. alginolyticus* [44,45]. These observations underscore a general trend: certain endolysins derived from vibriophages can exhibit broader lytic ranges than their corresponding phages, making them valuable tools against multiple strains or species within the Vibrio genus [36].

The specificity of endolysins toward their host bacteria highlights an evolutionary adaptation to degrade cell walls with distinct structural features. Endolysins typically contain specialized cell wall binding domains (CBDs) that recognize particular components, such as the glycine-rich peptidoglycan in *Staphylococcus aureus*, enabling the targeted lysis of *S. aureus* and closely related streptococci [46]. Similarly, Vibrio endolysins, such as those derived from LysVPp1, show lytic effects on several Vibrio species [42]. Some phage-derived endolysins, like LysAB2, initially designed to target *Acinetobacter baumannii*, and Art-175, a modified endolysin effective against *Pseudomonas aeruginosa*, incorporate cationic peptides that facilitate passage through the outer membrane, demonstrating potential to act on Gram-negative bacteria when adapted properly [47]. These findings suggest that for broader therapeutic applications, engineering strategies must enhance endolysin permeability to the outer membrane in Gram-negative aquaculture pathogens like *Vibrio* and *Tebacibaculum*, thus overcoming natural host specificity and expanding their antibacterial potential.

## 4. Materials and Methods

**Figure 4:**
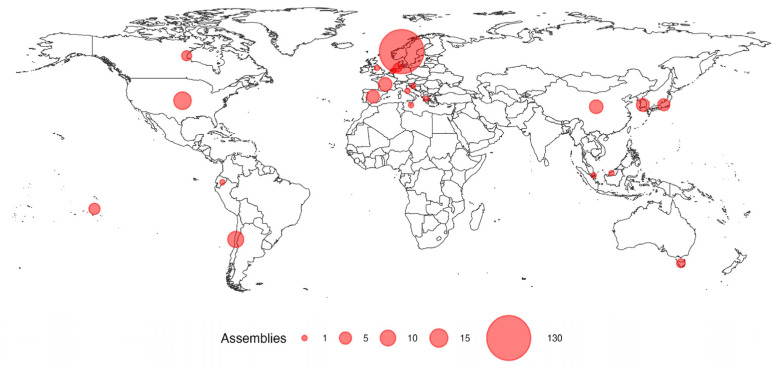
Geographical distribution of genomes/assemblies of *Tenacibaculum* species used in this study. The size of the circles is proportional to the number of assemblies. Genomic information was grouped by country from where the strains were isolated, considering the average latitude and longitude coordinates per country.

A total of 212 genomes/assemblies available in the NCBI repository were included in this study. The genomic information is derived from different sources, including strain type, isolates from aquaculture species, covering different parts of the world (Figure 4). The assemblies comprise strains of the species *T. aiptasiae* (5), *T. aquimarinum* (2), *T. bernardetii* (3), *T. dicentrarchi* (1), *T. discolor* (4), *T. finnmarkense* (129), *T. gallaicum* (2), *T. larymnensis* (1), *T. lutimaris* (2), *T. maritimum* (40), *T. mesophilum* (10), *T. ovolyticum* (4), *T*. *pacificus* (1), *T. retecalamus* (2), *T. singaporense* (4), and *T. todarodis* (2). The assemblies included in this study were filtered based on an average nucleotide identity (ANI) equal to or greater than 90%. The accession codes, host, country, and year of isolation for each genomic sequence used are included in Supplementary Table S3.

Genomic sequences were screened for the presence of prophage elements using the PHASTEST (PHAge Search Tool with Enhanced Sequence Translation) web server [48]. For those strains with complete genomes, the associated accession code was entered; in the case of whole genome shotgun (WGS) sequencing, the FASTA files containing the contigs were entered, and the checkbox was ticked to submit a file consisting of multiple separate contigs. PHASTEST classifies prophage regions into three categories according to a score assigned based on a comparison with the NCBI virus database. Depending on the score, the region is assigned to one of three categories: intact (score above 90), questionable (score between 70 and 90), and incomplete (score below 70). The score assignment is based on three methods. In the first one, if the number of a certain phage in the database is greater than or equal to 100% of the total number of CDS in the region, a total score of 150 is assigned; if this is not the case, the following methods are used. In the second method, a comparison is made similarly to the first, but the number of coding sequences (CDS) in the region is reduced by 50%. The phage with the highest likelihood for that region is identified as the primary candidate. Additionally, the percentage of proteins in the region matching the most probable phage is calculated and multiplied by 100 to generate a partial score. This is followed by comparing the length of the region with the most likely phage and multiplying this percentage by 50, which is then added to the partial score to calculate the final score. This approach also incorporates the identification of specific keywords related to phage components (e.g., capsid, head, integrase, plate, tail, fiber, coat, transposase, portal, terminase, protease, or lysin) to enhance the accuracy of phage predictions. Additionally, it is increased by 10 points if the size of the region is more than 30 Kb and by another 10 points if there are at least 40 proteins in the region. Finally, if all phage-related proteins and hypothetical proteins constitute more than 70% of the total proteins in the region, the score is increased by 10. As a final step, the scores from methods 2 and 3 are compared, and the highest score is chosen as the total score for the region.

The complete genomes of the most common phages found in the regions classified as intact prophages were compared with the genomes of the two phages related to *Tenacibaculum*, PTm5 (AP019525) and PTm1 (AP019524), using VICTOR [49], the Virus Classification and Tree Building Online Resource, freely available online from https://ggdc.dsmz.de/victor.php (accessed on 3 October 2024), a method for the genome-based phylogeny and classification of prokaryotic viruses. All pairwise comparisons of the amino acid sequences were conducted using the Genome BLAST Distance Phylogeny (GBDP) method [50] under settings recommended for prokaryotic viruses.

The amino acid sequence of the putative endolysin found in the intact prophage regions was first evaluated in the ProtParam tool (available at http://web.expasy.org/protparam/, accessed on 4 October 2024) to investigate the primary structure properties [51]. The prediction of transmembrane helices in the protein was evaluated in DeepTMHMM version 1.0 [52] (available at https://services.healthtech.dtu.dk/services/DeepTMHMM-1.0/, accessed on 4 October 2024). The InterPro tool version 102.0 [53] was used for a functional analysis of the putative endolysin. The endolysin sequence was also evaluated in Phyre2 version 2.0 [54] to identify the closest protein templates based on the predicted structure.

Genome assemblies of *Tenacibaculum* strains that presented prophage regions classified as intact were evaluated in the DefenseFinder tool version 0.1.0 [55] to discover the presence of antiphage defense systems.

## 5. Conclusions

This study identifies key prophage elements within *Tenacibaculum* genomes, with a notable concentration in *T. maritimum*, which may serve as promising candidates for phage therapy in combating tenacibaculosis in aquaculture. The stable, CHAP domain-containing endolysin found in intact prophage regions demonstrates structural compatibility with known lytic domains, suggesting it could effectively target *Tenacibaculum* cells. These findings support the feasibility of bacteriophage-based treatments as a sustainable alternative to antibiotics, addressing resistance concerns while paving the way for targeted, environmentally friendly disease management strategies in the aquaculture sector. In this context, genomic and metagenomic studies play a crucial role by uncovering deeper insights into phage–pathogen interactions, providing valuable opportunities to identify new control tools and further advance sustainable approaches to combating bacterial diseases.

## Figures and Tables

**Table 1:** Prophages identified in *Tenacibaculum* species assemblies using PHASTEST.

Species (Strain)	Region	Region Length	Completeness	Score	Total Proteins	Region Position	Most Common Phage	GC%
*T. maritimum* (TF4)	1	50 Kb	Intact	140	55	3079–53,132	Cellul_phi19:1	30.29
*T. maritimum* (FC)	1	15.8 Kb	Intact	100	22	13,262–29,093	Flavob_vB_FspM_pippi8_1	33.2
*T. maritimum* (NBRC 15946)	1	15.8 Kb	Intact	100	21	35,460–51,291	Flavob_vB_FspM_lotta8_1	33.22
*T. maritimum* (902)	1 2	21.5 Kb 11.5 Kb	Intact Incomplete	100 50	20 18	12,829–34,424 18,576–40,083	Cellul_phi19:1 Flavob_vB_FspM_lotta8_1	29.99 31.6
*T. maritimum* (JIP 32/91-4)	1	17.2 Kb	Questionable	80	12	31,735–48,966	Cellul_phi19:1	30.34
*T. maritimum* (P4-45)	1	22.2 Kb	Questionable	90	31	139–22,399	Flavob_vB_FspM_pippi8_1	34.09
*T. maritimum* (NVIB-2097)	1	11.3 Kb	Incomplete	50	18	1–11,355	Flavob_vB_FspM_lotta8_1	31.61
*T. maritimum* (NVIB-2096)	1	11.3 Kb	Incomplete	50	18	1–11,355	Flavob_vB_FspM_pippi8_1	31.61
*T. maritimum* (DPIF 89/0239-1)	1	11 Kb	Incomplete	50	16	74,959–86,020	Cellul_phi46:1	31.79
*T. mesophilum* (Mcav3-52)	1	38 Kb	Intact	110	45	360,314–398,338	Cellul_phi39:1	31.95
*T. mesophilum* (bac2)	1	12.7 Kb	Questionable	70	13	1,752,433–1,765,200	Strept_BRock	33.26
*T. mesophilum* (DSM 13764)	1	9.3 Kb	Questionable	70	9	1,339,122–1,348,490	Bacill_BCD7	33.93
*T. mesophilum* (DSM 13764)	1	9.3 Kb	Questionable	70	9	2,013,166–2,022,534	Strept_BRock	33.93
*T. mesophilum* (546.fasta)	1	9.3 Kb	Incomplete	60	9	31,278–40,645	Strept_BRock	33.85
*T. mesophilum* (XPcli2-G)	1	9.3 Kb	Incomplete	60	9	84,611–93,978	Bacill_BCD7	34
*T. mesophilum* (SSh1-16)	1	9.3 Kb	Incomplete	60	9	31,113–40,481	Bacill_BCD7	34
*T. pacificus* (P3-BQ1)	1	22 Kb	Intact	110	31	2,677,157–2,699,185	Flavob_vB_FspM_lotta8_1	30.2
*T. ovolyticum* (20-4135-2)	1	9.3 Kb	Questionable	80	9	3,744,277–3,753,604	Bacill_BCD7	31.89
*T. ovolyticum* (To-7Br)	1	9.3 Kb	Questionable	70	9	21,823–31,151	Strept_BRock	31.79
*T. ovolyticum* (da5A-8)	1	9.3 Kb	Questionable	70	9	80,624–89,952	Strept_BRock	31.76
*T. discolor* (LAR25)	1	7.4 Kb	Incomplete	60	8	256,719–264,196	Serrat_phiMAM1	32.23
*T. discolor* (IMLK18)	1	5.6 Kb	Incomplete	60	6	338,872–344,494	Synech_S_CBP3	30.82
*T. finnmarkense* (NVIB-3194)	1	16.7 Kb	Incomplete	40	22	3–16,707	Cellul_phiSM	30.55
*T. dicentrarchi* (TD3509T)	1	9.3 Kb	Incomplete	50	8	853,075–862,413	Flavob_vB_FspM_pippi8_1	33.5

## Data Availability

Data available on request.

## Supplementary Materials

The following supporting information can be downloaded at https://www.mdpi.com/article/10.3390/antibiotics13121184/s1, Figure S1: Results of the InterPro analysis of the putative endolysin; Figure S2: Predicted structure of the putative endolysin using Phyre2; Table S1: Phage-associated CDS; Table S2: Antiphage systems; Table S3: Assembly/genome data.

## References

[1] Mabrok M., Algammal A.M., Sivaramasamy E., Hetta H.F., Atwah B., Alghamdi S., Fawzy A., Avendaño-Herrera R., Rodkhum C. (2023). Tenacibaculosis Caused by *Tenacibaculum maritimum*: Updated Knowledge of This Marine Bacterial Fish Pathogen. Front. Cell. Infect. Microbiol..

[2] Nowlan J.P., Lumsden J.S., Russell S. (2020). Advancements in characterizing Tenacibaculum infections in Canada. Pathogens.

[3] Avendaño-Herrera R., Toranzo A.E., Magariños B. (2006). Tenacibaculosis Infection in Marine Fish Caused by Tenacibaculum maritimum: A Review. Dis. Aquat. Organ..

[4] Piñeiro-Vidal M., Gijón D., Zarza C., Santos Y. (2012). *Tenacibaculum dicentrarchi* sp. nov., a marine bacterium of the family Flavobacteriaceae isolated from European sea bass. Int. J. Syst. Evol. Microbiol..

[5] Piñeiro-Vidal M., Riaza A., Santos Y. (2008). Tenacibaculum discolor sp. nov. and *Tenacibaculum gallaicum* sp. nov., isolated from sole (*Solea senegalensis*) and turbot (*Psetta maxima*) culture systems. Int. J. Syst. Evol. Microbiol..

[6] Suzuki M., Nakagawa Y., Harayama S., Yamamoto S. (2001). Phylogenetic Analysis and Taxonomic Study of Marine Cytophaga-like Bacteria: Proposal for *Tenacibaculum* gen. nov. with *Tenacibaculum maritimum* comb. nov. and *Tenacibaculum ovolyticum* comb. nov., and Description of *Tenacibaculum mesophilum* sp. nov. and *Tenacibaculum amylolyticum* sp. nov. Int. J. Syst. Evol. Microbiol..

[7] Samsing F., Barnes A.C. (2024). The Rise of the Opportunists: What Are the Drivers of the Increase in Infectious Diseases Caused by Environmental and Commensal Bacteria?. Rev. Aquac..

[8] Spilsberg B., Nilsen H.K., Tavornpanich S., Gulla S., Jansen M.D., Lagesen K., Colquhoun D.J., Olsen A. (2022). Tenacibaculosis in Norwegian Atlantic Salmon (*Salmo salar*) Cage-Farmed in Cold Seawater Is Primarily Associated with *Tenacibaculum finnmarkense* Genomovar finnmarkense. J. Fish Dis..

[9] Servicio Nacional de Pesca y Acuicultura Informe con Antecedentes Sanitarios de Agua Dulce y Mar. https://www.sernapesca.cl/app/uploads/2024/09/Informe-Sanitario-ANO-2023.pdf.

[10] https://www.hipra.com/en/icthiovac-tm.

[11] Higuera G., Bastías R., Tsertsvadze G., Romero J., Espejo R.T. (2013). Recently discovered Vibrio anguillarum phages can protect against experimentally induced vibriosis in Atlantic salmon, Salmo salar. Aquaculture.

[12] Kalatzis P.G., Bastías R., Kokkari C., Katharios P. (2016). Isolation and Characterization of Two Lytic Bacteriophages, St2 and Grn1; Phage Therapy Application for Biological Control of Vibrio alginolyticus in Aquaculture Live Feeds. PLoS ONE.

[13] Katharios P., Kalatzis P.G., Kokkari C., Sarropoulou E., Middelboe M. (2017). Isolation and characterization of a N4-like lytic bacteriophage infecting Vibrio splendidus, a pathogen of fish and bivalves. PLoS ONE.

[14] https://stim.uk/r-d/bacteriophages/.

[15] Tsertou M.I., Triga A., Droubogiannis S., Kokkari C., Anasi G., Katharios P. (2023). Isolation and Characterization of a Novel Tenacibaculum Species and a Corresponding Bacteriophage from a Mediterranean Fish Hatchery: Description of Tenacibaculum larymnensis sp. nov. and Tenacibaculum Phage Larrie. Front. Microbiol..

[16] de Jonge P.A., Nobrega F.L., Brouns S.J.J., Dutilh B.E. (2019). Molecular and Evolutionary Determinants of Bacteriophage Host Range. Trends Microbiol..

[17] Bobay L.M., Rocha E.P.C., Touchon M. (2013). The Adaptation of Temperate Bacteriophages to Their Host Genomes. Mol. Biol. Evol..

[18] Abad L., Gauthier C.H., Florian I., Jacobs-Sera D., Hatfull G.F. (2023). The Heterogeneous and Diverse Population of Prophages in *Mycobacterium* Genomes. mSystems.

[19] Sharma V., Hünnefeld M., Luthe T., Frunzke J. (2023). Systematic Analysis of Prophage Elements in Actinobacterial Genomes Reveals a Remarkable Phylogenetic Diversity. Sci. Rep..

[20] Butala M., Dragoš A. (2023). Unique Relationships between Phages and Endospore-Forming Hosts. Trends Microbiol..

[21] Wang Z., Peng X., Hülpüsch C., Khan Mirzaei M., Reiger M., Traidl-Hoffmann C., Deng L., Schloter M. (2024). Distinct Prophage Gene Profiles of Staphylococcus aureus Strains from Atopic Dermatitis Patients and Healthy Individuals. Microbiol. Spectr..

[22] Szafrański S.P., Kilian M., Yang I., Bei der Wieden G., Winkel A., Hegermann J., Stiesch M., Szafrański S.P., Kilian M., Yang I. (2019). Diversity Patterns of Bacteriophages Infecting Aggregatibacter and Haemophilus Species across Clades and Niches. ISME J..

[23] Brüssow H., Canchaya C., Hardt W.D. (2004). Phages and the Evolution of Bacterial Pathogens: From Genomic Rearrangements to Lysogenic Conversion. Microbiol. Mol. Biol. Rev..

[24] Castillo D., Kauffman K., Hussain F., Kalatzis P., Rørbo N., Polz M.F., Middelboe M. (2018). Widespread Distribution of Prophage-Encoded Virulence Factors in Marine *Vibrio* Communities. Sci. Rep..

[25] Rohde C., Resch G., Pirnay J.P., Blasdel B.G., Debarbieux L., Gelman D., Górski A., Hazan R., Huys I., Kakabadze E. (2018). Expert Opinion on Three Phage Therapy-Related Topics: Bacterial Phage Resistance, Phage Training, and Prophages in Bacterial Production Strains. Viruses.

[26] León M., Bastías R. (2015). Virulence Reduction in Bacteriophage-Resistant Bacteria. Front. Microbiol..

[27] León M., Kokkari C., García K., Castillo D., Katharios P., Bastías R. (2019). Diversification of *Vibrio anguillarum* Driven by the Bacteriophage CHOED. Front. Microbiol..

[28] Doron S., Melamed S., Ofir G., Leavitt A., Lopatina A., Keren M., Amitai G., Sorek R. (2018). Systematic discovery of antiphage defense systems in the microbial pangenome. Science.

[29] Wu Y., Garushyants S.K., van den Hurk A., Aparicio-Maldonado C., Kushwaha S.K., King C.M., Ou Y., Todeschini T.C., Clokie M.R., Millard A.D. (2024). Bacterial Defense Systems Exhibit Synergistic Anti-Phage Activity. Cell Host Microbe.

[30] Nasrullah H.A., Ahmed S., Rasool M., Shah A.J. (2022). DNA Methylation across the Tree of Life, from Micro to Macro-Organism. Bioengineered.

[31] Millman A., Melamed S., Leavitt A., Doron S., Bernheim A., Hör J., Garb J., Bechon N., Brandis A., Lopatina A. (2022). An Expanded Arsenal of Immune Systems That Protect Bacteria from Phages. Cell Host Microbe.

[32] Brouns S.J.J., Jore M.M., Lundgren M., Westra E.R., Slijkhuis R.J.H., Snijders A.P.L., Dickman M.J., Makarova K.S., Koonin E.V., van der Oost J. (2008). Small CRISPR RNAs Guide Antiviral Defense in Prokaryotes. Science.

[33] Zhang T., Cepauskas A., Nadieina A., Thureau A., Coppieters ‘t Wallant K., Martens C., Lim D.C., Garcia-Pino A., Laub M.T. (2024). A Bacterial Immunity Protein Directly Senses Two Disparate Phage Proteins. Nature.

[34] de Freitas Almeida G.M., Hoikkala V., Ravantti J., Rantanen N., Sundberg L.R. (2022). Mucin Induces CRISPR-Cas Defense in an Opportunistic Pathogen. Nat. Commun..

[35] Almeida G.M.F., Laanto E., Ashrafi R., Sundberg L.-R. (2019). Bacteriophage adherence to mucus mediates preventive protection against pathogenic bacteria. mBio.

[36] Romero J., Blas-Chumacero S., Urzúa V., Villasante A., Opazo R., Gajardo F., Miranda C.D., Rojas R. (2024). Lysin and Lytic Phages Reduce Vibrio Counts in Live Feed and Fish Larvae. Microorganisms.

[37] Cheng G., Hao H., Xie S., Wang X., Dai M., Huang L., Yuan Z. (2014). Antibiotic alternatives: The substitution of antibiotics in animal husbandry?. Front. Microbiol..

[38] Nachimuthu R., Madurantakam Royam M., Manohar P., Leptihn S. (2021). Application of bacteriophages and endolysins in aquaculture as a biocontrol measure. Biological Control.

[39] Horgan M., O’Flynn G., Garry J., Cooney J., Coffey A., Fitzgerald G., Ross P., McAuliffe O. (2009). Phage Lysin LysK Can Be Truncated to Its CHAP Domain and Retain Lytic Activity against Live Antibiotic-Resistant Staphylococci. Appl. Environ. Microbiol..

[40] Hjelm L.C., Nilvebrant J., Nygren P.-Å., Nilsson A.S., Seijsing J. (2019). Lysis of Staphylococcal Cells by Modular Lysin Domains Linked via an Interaction Bridge. Front. Microbiol..

[41] Li M., Jin Y., Lin H., Wang J., Jiang X. (2018). Complete Genome of a Novel Lytic Vibrio parahaemolyticus Phage VPp1 and Characterization of Its Endolysin for Antibacterial Activities. J. Food Prot..

[42] Lal T.M., Sano M., Ransangan J. (2016). Genome characterization of a novel vibriophage VpKK5 (Siphoviridae) specific to fish pathogenic strain of *Vibrio parahaemolyticus*. J. Basic Microbiol..

[43] Matamp N., Bhat S.G. (2019). Phage Endolysins as Potential Antimicrobials against Multidrug Resistant *Vibrio alginolyticus* and *Vibrio parahaemolyticus*: Current Status of Research and Challenges Ahead. Microorganisms.

[44] Ning H., Cong Y., Lin H., Wang J. (2021). Development of cationic peptide chimeric lysins based on phage lysin Lysqdvp001 and their antibacterial effects against *Vibrio parahaemolyticus*: A preliminary study. Int. J. Food Microbiol..

[45] Wang L., Ju X., Cong Y., Lin H., Wang J. (2022). A Single Catalytic Endolysin Domain Plychap001: Characterization and Application to Control *Vibrio parahaemolyticus* and Its Biofilm Directly. Foods.

[46] Schmelcher M., Donovan D.M., Loessner M.J. (2012). Bacteriophage Endolysins as Novel Antimicrobials. Future Microbiol..

[47] Briers Y., Walmagh M., Van Puyenbroeck V., Cornelissen A., Cenens W., Aertsen A., Oliveira H., Azeredo J., Verween G., Pirnay J.-P. (2014). Engineered Endolysin-Based “Artilysins” to Combat Multidrug-Resistant Gram-Negative Pathogens. mBio.

[48] Wishart D.S., Han S., Saha S., Oler E., Peters H., Grant J., Stothard P., Gautam V. (2023). PHASTEST: Faster than PHASTER, Better than PHAST. Nucleic Acids Res..

[49] Meier-Kolthoff J.P., Göker M. (2017). VICTOR: Genome-Based Phylogeny and Classification of Prokaryotic Viruses. Bioinformatics.

[50] Meier-Kolthoff J.P., Auch A.F., Klenk H.P., Göker M. (2013). Genome Sequence-Based Species Delimitation with Confidence Intervals and Improved Distance Functions. BMC Bioinform..

[51] Gasteiger E., Hoogland C., Gattiker A., Duvaud S., Wilkins M.R., Appel R.D., Bairoch A., Walker J.M. (2005). Protein Identification and Analysis Tools on the ExPASy Server. The Proteomics Protocols Handbook.

[52] Hallgren J., Tsirigos K.D., Pedersen M.D., Almagro Armenteros J.J., Marcatili P., Nielsen H., Krogh A., Winther O. DeepTMHMM Predicts Alpha and Beta Transmembrane Proteins Using Deep Neural Networks. bioRxiv.

[53] Paysan-Lafosse T., Blum M., Chuguransky S., Grego T., Pinto B.L., Salazar G.A., Bileschi M.L., Bork P., Bridge A., Colwell L. (2022). InterPro in 2022. Nucleic Acids Res..

[54] Kelley L., Mezulis S., Yates C., Wass M.N., Sternberg M.J.E. (2015). The Phyre2 Web Portal for Protein Modeling, Prediction and Analysis. Nat. Protoc..

[55] Tesson F., Hervé A., Mordret E., Touchon M., D’humières C., Cury J., Bernheim A. (2022). Systematic and Quantitative View of the Antiviral Arsenal of Prokaryotes. Nat. Commun..

