# Supplementary material for: Unveiling Hidden Allies: In Silico Discovery of Prophages in Tenacibaculum Species
Source: Antibiotics (Basel). 2024 Dec 5;13(12):1184. doi: 10.3390/antibiotics13121184 (PMC11672841; doi:10.3390/antibiotics13121184)
Supplement: Supplementary file 1 [file antibiotics-13-01184-s001.zip › antibiotics-3315354-Supplementary Materials Figures.pdf]

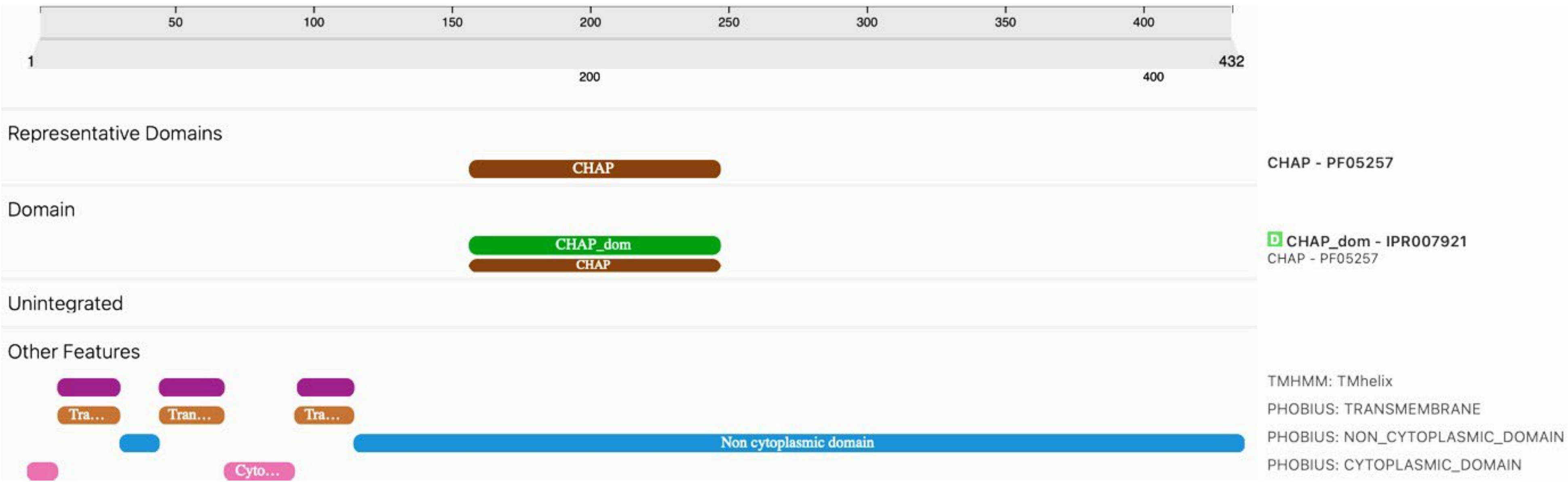

Figure S1. Results of the InterPro analysis of the putative endolysin.

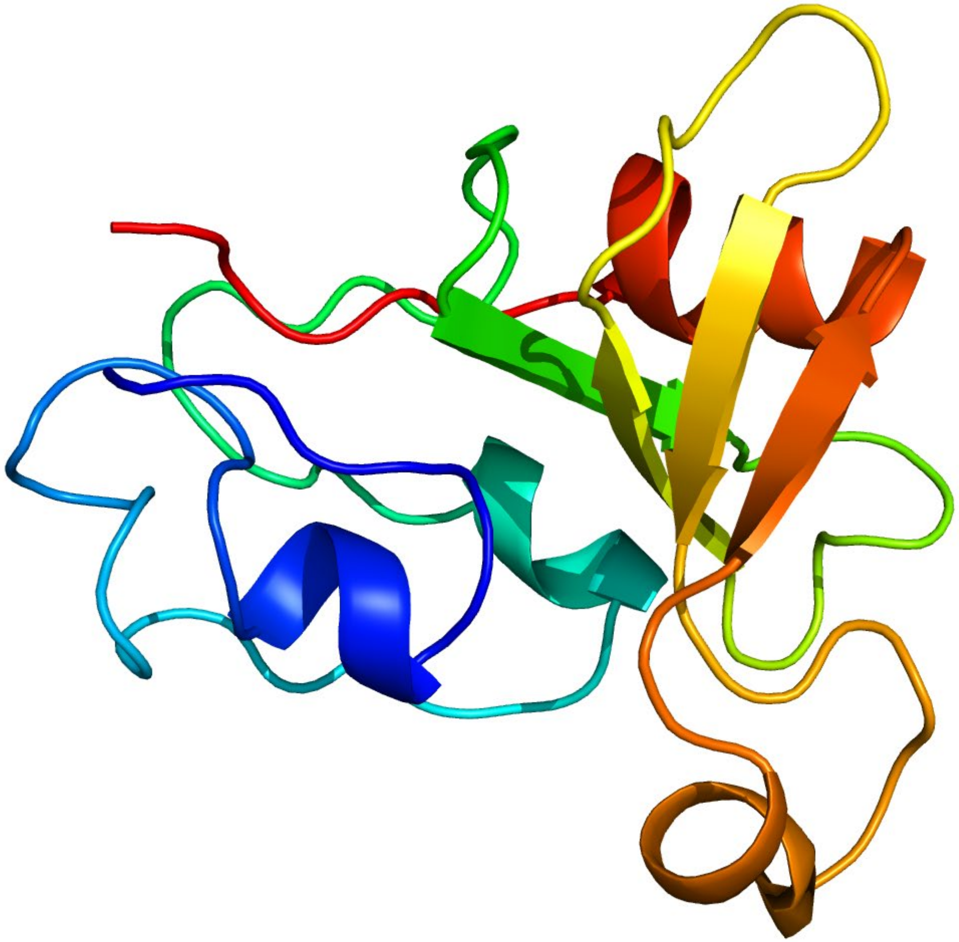

Figure S2. Predicted structure of putative endolysin using Phyre2.
